# Emitter Location Using Frequency Difference of Arrival Measurements Only

**DOI:** 10.3390/s22249642

**Published:** 2022-12-09

**Authors:** Mohamed Khalaf-Allah

**Affiliations:** Electrical Smart City Systems, Friedrich-Alexander-Universität Erlangen-Nürnberg, 91058 Erlangen, Germany; mohamed.khalaf.allah@fau.de; Tel.: +49-9131-85-20987

**Keywords:** emitter location, geolocation, frequency difference of arrival (FDoA), grid-based location estimation, sample-based location estimation

## Abstract

It is desirable to enable emitter location using frequency difference of arrival (FDoA) measurements only, since many signals are characterized by coarse range resolution and fine Doppler resolution. For instance, while using the cross-ambiguity function (CAF) to measure the time difference of arrival (TDoA) and the FDoA of a narrowband signal, it is difficult to obtain accurate TDoA measurements because the Doppler resolution is higher than the range resolution. Grid-based and sample-based algorithms are developed to solve the two-dimensional (2D) emitter location problem, where the solution space is approximated, respectively, by generating deterministic and random emitter location candidates. Simulation results corroborate the viability of both non-iterative algorithms to estimate the emitter location using a single-time snapshot of FDoA measurements only, without any prior location information or any knowledge about the distribution of measurement errors. The achieved accuracies are sufficient for early warning purposes, preparing defenses, and cueing more accurate location sensors by directing additional surveillance resources.

## 1. Introduction

Emitter location is desirable or even necessary in both civilian and military applications. Therefore, emitter (also target or source) location is the focus of considerable research efforts due to its importance in microphone arrays [1,2], sonar [3], radar [4], and electronic warfare (EW) [5] applications. In emergencies, it is of paramount importance to be able to determine the location of the broadcasted distress radio signals to direct rescue as accurately and quickly as possible. The angle of arrival (AoA) [5]; time difference of arrival (TDoA) [6]; frequency difference of arrival (FDoA) [7,8,9], also known as differential Doppler (DD); and differential Doppler rate (DDR) [10] measurements of the emitted signals are typical parameters for location of noncooperative emitting sources.

The AoA, TDoA, and FDoA location parameters can be utilized in isolation or jointly by combining two techniques only [11] or by combining two techniques with an additional method [10] in one approach. The pairing of TDoA and FDoA [12,13] high-precision emitter location techniques is the most common arrangement discussed in the literature because both require simple sensors at the receiver stations, whereas the AoA technique requires more complex receivers at each direction finding (DF) station [14]. DF requires bulky and expensive directional antennas, and the accuracy is severely impacted by the emitter motion, as well as the duration of signal emission. TDoA/FDoA techniques are not subject to these limiting factors [15]. For example, it is difficult to equip an unmanned aircraft system (UAS) with a directional antenna capable of receiving the very high frequency (VHF) band due to the size, weight, and power (SWaP) constraints, whereas a smaller non-directional antenna can be employed instead when using the TDoA/FDoA geolocation technique [15]. The performance of TDoA/FDoA is dependent on the accuracy of the timing synchronization [16] of the correlated signals and not the time length of the signal emission. Therefore, TDoA/FDoA techniques are not impaired if the duration of the emitted signals is extremely short. In contrast, DF techniques require a longer duration of the emitted signals to achieve a comparable performance [15].

Geolocation accuracy is impacted by the signal-to-noise ratio (SNR) level. In general, the greater the range to the emitter, the lower the SNR and the less accurate the geolocation performance, and vice versa. The performance of DF techniques degrades linearly with the range to the emitter, whereas TDoA/FDoA approaches are less dependent on the SNR level (and hence the range), since the SNR is only part of the overall error contribution from the Cramér–Rao lower bound (CRLB), due to taking advantage of the measured differences in time and frequency of arrival [15]. Therefore, the accuracy of TDoA/FDoA approaches is less impacted by the range to the emitter than DF techniques, provided the SNR level is adequate for performing measurements.

Emitter location using the above measurement techniques is a nonlinear estimation problem and, therefore, is a nontrivial task. Although the FDoA technique is well studied and understood, it is less frequently used as a standalone solution for emitter location than the TDoA or AoA techniques. FDoA-based emitter location, in particular, is highly nonlinear and, therefore, the solution estimation problem is ill-conditioned and sensitive to noise [17]. The FDoA technique also requires that either the emitter or the receiver/sensor stations (more commonly) be moving to generate the DD needed for the FDoA measurement. Uncertainties in the sensor’s velocity, i.e., magnitude and direction of motion, make obtaining accurate frequency measurements for FDoA more difficult.

Signals of all kinds, including radar signals, can be represented as a function of time or frequency, and both expressions are related via the Fourier transform, where repetitive properties in one domain appear differently in the other [18]. The cross-ambiguity function (CAF), which conventionally determines the performance of a waveform, represents the response of sensors/receivers to a point emitting source, as a function of time delay and Doppler shift. Since many signals are characterized by coarse range, i.e., TDoA, resolution, and fine Doppler resolution when using the CAF to measure the TDoA and FDoA [19], it is desirable to enable emitter location using FDoA measurements only in case of, e.g., a narrowband signal [17,19], because the Doppler resolution is higher than the range resolution and, therefore, it is difficult to obtain accurate TDoA measurements. The radar ambiguity function also confirms the inverse relationship between Doppler and range resolutions [20]. Thus, operators of radars can control the type of transmitted signals and, therefore, the development of FDoA-based emitter location algorithms is especially valuable and beneficial to extend the capabilities of EW systems to process diverse sets of waveforms [17], since TDoA-based emitter location is well studied [21,22]. Thus, new methods to measure the FDoA only, instead of the joint TDoA/FDoA, are also motivated in, e.g., [23]. The focus of this study is on emitter location once FDoA measurements are obtained. Signal processing techniques used to extract FDoA measurements are beyond the scope of this study.

Geolocation of a stationary emitter by moving sensors using FDoA measurements only is a typical application scenario. It was accomplished in [19] by the linear-correction least-squares estimation approach, which is similar to the method reported in [24]. The problem was formulated in [17] as a system of polynomials whose solution corresponds to the emitter’s location. Therefore, numerical algebraic geometry methods were utilized to solve the problem. Further research work has been reported in [25,26,27,28,29,30,31]. Another application scenario considers a moving emitter and stationary sensors, in which the emitter’s velocity is an unknown parameter in addition to its location. This scenario was addressed in, e.g., [32,33,34,35,36,37,38,39,40,41]. The FDoA-based emitter location problem is ill-posed [17]. Thus, it is difficult to distinguish the correct solution from several possible ones. Therefore, iterative approaches [42,43,44] can converge to a wrong solution if the initial guess is not close enough to the true emitter location, which is difficult to define, making it difficult to obtain a good initial guess in many practical situations. Moreover, FDoA-based emitter location is sensitive to noise. Thus, small perturbations can cause highly inaccurate solutions. Both drawbacks are due to the high nonlinearity of the problem. Other solution approaches include closed-form algorithms [29,40,41,45,46], numerical search methods [25,26,27,28,33,34,35,47], and semidefinite relaxation [29,40,41,48,49].

In this article, the 2D emitter location problem is addressed. Grid-based and sample-based algorithms are developed to solve the problem, where the solution space is approximated, respectively, by generating deterministic and random emitter location candidates. The performance is investigated by varying several parameters and evaluated by the CRLB benchmarking. Thus, the main contribution is to demonstrate the viability of both non-iterative algorithms to estimate the emitter location using a single-time snapshot of FDoA measurements only, without any prior location information or any knowledge about the distribution of measurement errors.

The remainder of the article is organized as follows: problem formulation and the performance metric are stated in Section 2. Solution algorithms are developed in Section 3. Simulation results are presented and discussed in Section 4, and the study is concluded in Section 5.

## 2. Problem Statement

Emitting sources are termed targets and emitters in the radar and the electronic support (ES) communities, respectively. The term tracks, common to the lexicon of both communities, represent the system’s estimate of emitting sources [50]. FDoA is considered in combination with TDoA and not in isolation in most formulations available in the literature, mainly to reduce the number of sensors required and to take advantage of the often complementary error characteristics. The FDoA technique is less widely adopted than the TDoA approach due to the additional inherent complexity and issues related to obtaining sufficiently accurate frequency measurements for emitter location [14].

**Figure 1:**
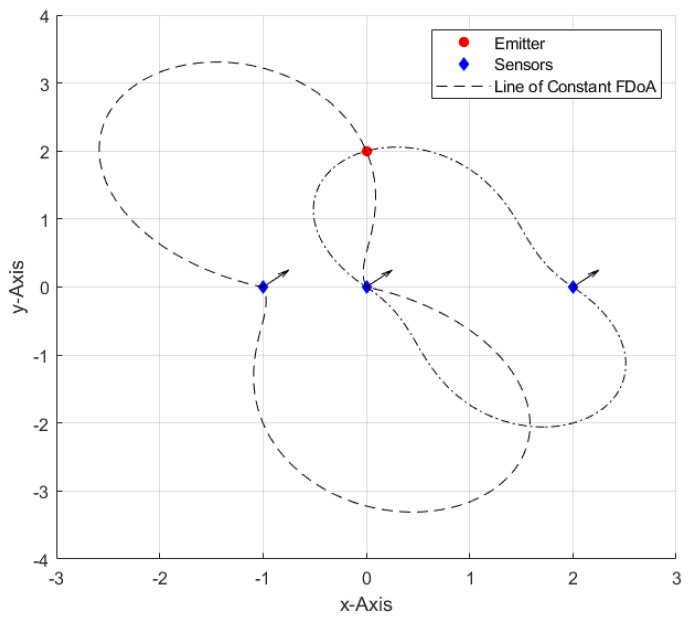
Lines of constant frequency difference of arrival (FDoA) with a stationary emitter and three sensors moving toward the indicated direction.

In the context of FDoA measurements only, each of the moving sensors measures the emitter’s exact carrier frequency slightly differently due to the Doppler shift, which is proportional to the relative velocity of the emitter as observed by each sensor. The FDoA of a sensor pair generates a curve of possible emitter locations. The intersection of two or more curves resolves the location ambiguity. Figure 1, adopted from [14], illustrates iso-Doppler contours or lines of constant FDoA in the 2D space. Whereas the possible TDoA-based emitter location is limited to a hyperbolic curve or the surface of a hyperboloid in the 2D and three-dimensional (3D) spaces, respectively, the geometric interpretation of the FDoA-based emitter location is not as simple. The emitter’s actual carrier frequency cannot be directly estimated with FDoA. Nevertheless, the emitter location can be accomplished by comparing the obtained FDoA measurements [14]. FDoA measurements between each pair of sensors can be obtained by estimating the emitter’s carrier frequency at each sensor to get the differences, or by direct comparison of the received signals, which is more accurate, but requires the transmission of raw sensor data and, therefore, signal compression would be required to minimize communication burdens [14].

### 2.1. Formulation

The adopted signal model assumes that the transmitted signal, s(t), propagates at the speed of light, c, and experiences attenuation, time delay (proportional to the range between the emitter and receiver), and Doppler shift (proportional to the relative velocity between the emitter and receiver). Thus, the received signal at the *i*th sensor, $y_{i}\left(t\right)$, is defined [14]:

(1) $$y_{i}\left(t\right)=\alpha_{i}s\left(t-\tau_{i}\right)e^{j2\pi f_{i}\left(t-\tau_{i}\right)}+n_{i}\left(t\right)$$

where $\alpha_{i}$ is the attenuation factor, $\tau_{i}$ is the time delay, $f_{i}$ is the Doppler shift, and $n_{i}\left(t\right)$ is the noise term.

The range, $R_{i}\left(x\right)$, from the emitter to the *i*th sensor and the corresponding range rate, ${\dot{R}}_{i}\left(x,v\right)$, i.e., change in range over time, are defined:

(2) $$R_{i}\left(x\right)=\|x-x_{i}\|$$

(3) $${\dot{R}}_{i}\left(x,v\right)=\frac{{\left(v-v_{i}\right)}^{T}\left(x-x_{i}\right)}{R_{i}\left(x\right)}$$

where x and v are the emitter’s true position and velocity, respectively, and $x_{i}$ and $v_{i}$ are the *i*th sensor’s true position and velocity, respectively. The time delay, $\tau_{i}$, and Doppler shift, $f_{i}$, are, thus, defined as $\tau_{i}=\frac{R_{i}}{c}$ and $f_{i}=\frac{f_{0}}{c}{\dot{R}}_{i}$, where $f_{0}$ is the true center carrier frequency of the emitted signal, which may be changed intentionally and/or may contain uncertainties due to, e.g., instability of the electronics [51]. However, $f_{0}$ is assumed to have a fixed known value for the sake of simplicity.

The frequency difference between any two sensors, m and n, $f_{m,n}\left(x,v\right)$, is given as:

(4) $$f_{m,n}\left(x,v\right)=f_{m}\left(x,v\right)-f_{n}\left(x,v\right)=\frac{f_{0}}{c}\left[\frac{{\left(v_{m}-v\right)}^{T}\left(x-x_{m}\right)}{\left\|x-x_{m}\right\|}-\frac{{\left(v_{n}-v\right)}^{T}\left(x-x_{n}\right)}{\left\|x-x_{n}\right\|}\right]$$

The range rate difference between the *m*th and *n*th sensors, ${\dot{R}}_{m,n}\left(x,v\right)$, is, thus, computed:

(5) $${\dot{R}}_{m,n}\left(x,v\right)=\frac{c}{f_{0}}f_{m,n}\left(x,v\right)=\left[\frac{{\left(v_{m}-v\right)}^{T}\left(x-x_{m}\right)}{\left\|x-x_{m}\right\|}-\frac{{\left(v_{n}-v\right)}^{T}\left(x-x_{n}\right)}{\left\|x-x_{n}\right\|}\right]$$

Assuming a stationary emitter, i.e., v=0, Equation (5) is rewritten:

(6) $${\dot{R}}_{m,n}\left(x\right)=\left[\frac{v_{m}^{T}\left(x-x_{m}\right)}{\left\|x-x_{m}\right\|}-\frac{v_{n}^{T}\left(x-x_{n}\right)}{\left\|x-x_{n}\right\|}\right]$$

At least two range rate difference measurements, i.e., using three sensors and three range rate difference measurements, i.e., using four sensors, are required to estimate a stationary emitter’s location, x, in the 2D and 3D spaces, respectively. If the emitter is not stationary, the emitter’s velocity, v, then represents an additional unknown variable and, therefore, additional sensors or additional measurement types, e.g., TDoA or AoA, would be required to estimate v in addition to x.

Without loss of generality, the first sensor among N sensors, where N≥3, is considered as the common reference, i.e., the master, sensor. The vector form of the range rate differences, $\dot{r}\left(x\right)$, between sensor 1 and sensors 2 through N is written:

(7) $$\dot{r}\left(x\right)={\left[{\dot{R}}_{2,1}\left(x\right),\;{\dot{R}}_{3,1}\left(x\right),\ldots ,{\dot{R}}_{N,1}\left(x\right)\right]}^{T}$$

The vector form of the noisy range rate difference measurements, $\dot{\rho }\left(x\right)$, is given as:

(8) $$\dot{\rho }\left(x\right)=\dot{r}\left(x\right)+n$$

where n is a zero-mean Gaussian distributed random vector with the measurement error covariance matrix, $C_{\dot{\rho }}$, and all frequency measurements are assumed as independent. Thus, the probability density function (PDF), $f_{x}\left(\dot{\rho }\right)$, is written:

(9) $$f_{x}\left(\dot{\rho }\right)={\left(2\pi \right)}^{-\frac{\left(N-1\right)}{2}}{\left|C_{\dot{\rho }}\right|}^{-\frac{1}{2}}e^{-\frac{1}{2}{\left(\dot{\rho }-\dot{r}\left(x\right)\right)}^{T}C_{\dot{\rho }}^{-1}\left(\dot{\rho }-\dot{r}\left(x\right)\right)}$$

and the log-likelihood function, $\ell \left(x|\dot{\rho }\right)$, is given as:

(10) $$\ell \left(x|\dot{\rho }\right)=-\frac{1}{2}{\left(\dot{\rho }-\dot{r}\left(x\right)\right)}^{T}C_{\dot{\rho }}^{-1}\left(\dot{\rho }-\dot{r}\left(x\right)\right)$$

The problem, thus, is to estimate the location, x, of a stationary emitter given the noisy range rate difference measurements, $\dot{\rho }\left(x\right)$, and using the known sensors’ positions, $x_{i}$, and velocities, $v_{i}$, i=1,2,…,N, which, in turn, might contain uncertainties.

### 2.2. Performance Metric

The CRLB is used as the statistical bound on performance, which provides a lower bound on the mean-square error (MSE) that can be attained by an unbiased estimator. Therefore, the CRLB is a lower bound on the elements of the estimation error covariance matrix, $C_{\hat{x}}$, for an unbiased estimate, $\hat{x}$, i.e., $\mathrm{CRLB}\leq C_{\hat{x}}$. Thus, the square root of the CRLB is a lower bound on the root-mean-square error (RMSE) or equivalently the standard deviation, i.e., $\sqrt{\mathrm{CRLB}}\leq \mathrm{RMSE}$. Since the RMSE can be scaled for different confidence intervals, the CRLB can equivalently be scaled to show the bounds on various confidence intervals [14]. The CRLB is given by the inverse of the Fisher information matrix (FIM), F(x), which is computed in the general Gaussian case:

(11) $$F\left(x\right)=J\left(x\right)C_{\dot{\rho }}^{-1}J^{T}\left(x\right)$$

where J(x) is the Jacobian matrix of $\dot{r}\left(x\right)$, defined:

(12) $$J\left(x\right)=\left[\nabla_{x}{\dot{R}}_{2,1}\left(x\right),\;\nabla_{x}{\dot{R}}_{3,1}\left(x\right),\ldots ,\nabla_{x}{\dot{R}}_{N,1}\left(x\right)\right]$$

where $\nabla_{x}{\dot{R}}_{N,1}\left(x\right)$ is the gradient of the range rate difference to the emitter between the first sensor (reference sensor) and sensors 2 through N, and is computed [14]:

(13) $$\nabla_{x}{\dot{R}}_{N,1}\left(x\right)=\left(I-\frac{\left(x-x_{N}\right){\left(x-x_{N}\right)}^{T}}{\left\|x-x_{N}{}^{2}\right\|}\right)\frac{v_{N}}{\left\|x_{N}-x\right\|}-\left(I-\frac{\left(x-x_{1}\right){\left(x-x_{1}\right)}^{T}}{\left\|x-x_{1}{}^{2}\right\|}\right)\frac{v_{1}}{\left\|x_{1}-x\right\|}$$

Thus, $C_{\hat{x}}$ is bounded as:

(14) $$C_{\hat{x}}\geq {\left[J\left(x\right)C_{\dot{\rho }}^{-1}J^{T}\left(x\right)\right]}^{-1}$$

Assuming Gaussian FDoA measurements is convenient to derive the CRLB. However, it is not strictly accurate because whereas the measurements are Gaussian when projected onto spatial coordinates, they are non-Gaussian within the observation space [52], since $\dot{r}\left(x\right)$ is nonlinear. Therefore, appropriate estimation requires nonlinear information fusion of the FDoA measurements by, e.g., a Gaussian measurement mixture algorithm [52]. Nevertheless, the CRLB will be used for benchmarking, despite its acknowledged shortcomings, due to its ease of derivation.

## 3. Solution Algorithms

In this section, the solution to the 2D stationary emitter location problem using FDoA measurements only is developed. Direct evaluation of the highly nonlinear Equation (8) to estimate the emitter location is difficult. Feasible solutions include grid-based and sample-based calculations of the probability of the emitter occupying various locations, where the accuracy is limited by the grid resolution and number of samples, respectively. Therefore, the grid resolution and number of samples should be properly determined to reduce the computational burdens without sacrificing accuracy. The suggested solutions provide implementable algorithms that approximate the solution space, i.e., all possible emitter locations, by a finite number of emitter location candidates, which are generated deterministically or randomly by the grid-based and sample-based algorithms, respectively. A single value, i.e., weight, is assigned to each location candidate to represent the probability of the emitter occupying that location given the FDoA measurements in addition to the locations and velocities of the involved sensors.

**Figure 2:**
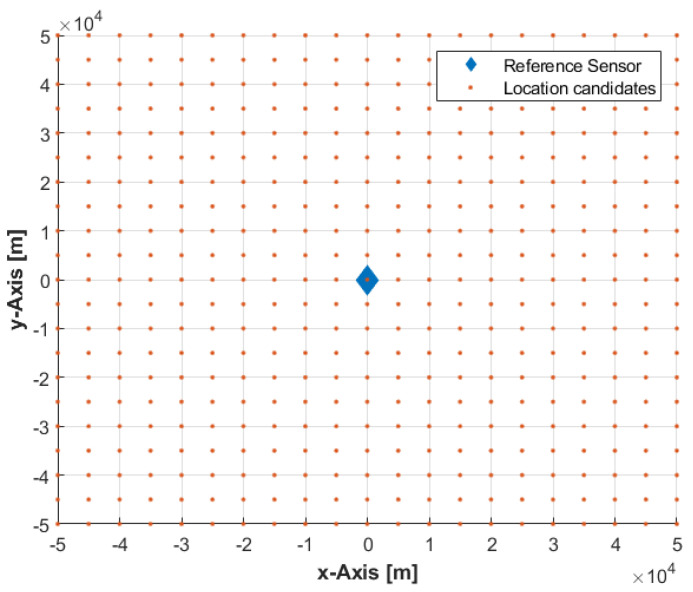
The solution space is approximated by a grid of emitter location candidates corresponding to the reference sensor coverage.

In the grid-based algorithm, the solution space, S, is approximated at any time instant by a grid of location/position coordinates over an area corresponding to the reference sensor coverage (see Figure 2), and is mathematically represented as:

(15) $$S\approx {\left\{s_{j},w_{j}\right\}}_{j=1:p}$$

where $s_{j}$ is the *j*th location candidate; $w_{j}$ is a probability value that determines the weight, i.e., importance, of $s_{j}$; and p is the total number of the grid locations, i.e., the set of location candidates.

The grid-based algorithm calculates the probability that the emitter lies in each grid location given the FDoA measurements in addition to the known locations and velocities of the involved sensors, which represents the discrete conditional PDF of the emitter location. The weight, $w_{j}$, is computed:

(16) $$w_{j}=1/{\left\|\dot{\rho }\left(x\right)-\dot{q}(s_{j})\right\|}^{2}$$

where $\dot{q}\left(s_{j}\right)$ is the set of range rate differences at the location candidate, $s_{j}$, and is given as:

(17) $$\dot{q}\left(s_{j}\right)={\left[{\dot{R}}_{2,1}\left(s_{j}\right),\;{\dot{R}}_{3,1}\left(s_{j}\right),\ldots ,{\dot{R}}_{N,1}\left(s_{j}\right)\right]}^{T}$$

The range rate difference between the reference sensor, i.e., sensor 1, and any of the remaining sensors, i.e., sensors 2 through N, is written:

(18) $${\dot{R}}_{N,1}\left(s_{j}\right)=\left[\frac{v_{N}^{T}\left(s_{j}-x_{N}\right)}{\left\|s_{j}-x_{N}\right\|}-\frac{v_{1}^{T}\left(s_{j}-x_{1}\right)}{\left\|s_{j}-x_{1}\right\|}\right]$$

Thus, the weight, $w_{j}$, of each emitter location candidate, $s_{j}$, is inversely proportional to the similarity metric used, which is the squared distance between $\dot{\rho }\left(x\right)$ and $\dot{q}\left(s_{j}\right)$. The value of $w_{j}$, in Equation (16), is normalized to make the sum of all weights equal unity, i.e., $\sum_{j=1}^{p}w_{j}=1$, so that S represents a valid probability distribution. The emitter location estimate, $\hat{x}$, is, thus, the location candidate with the highest assigned weight, and is expressed:

(19) $$\hat{x}=\arg\max_{s_{j}}{\left\{s_{j},w_{j}\right\}}_{j=1:p}$$

Therefore, normalization of the weights is not a crucial issue for practical algorithm implementation.

**Figure 3:**
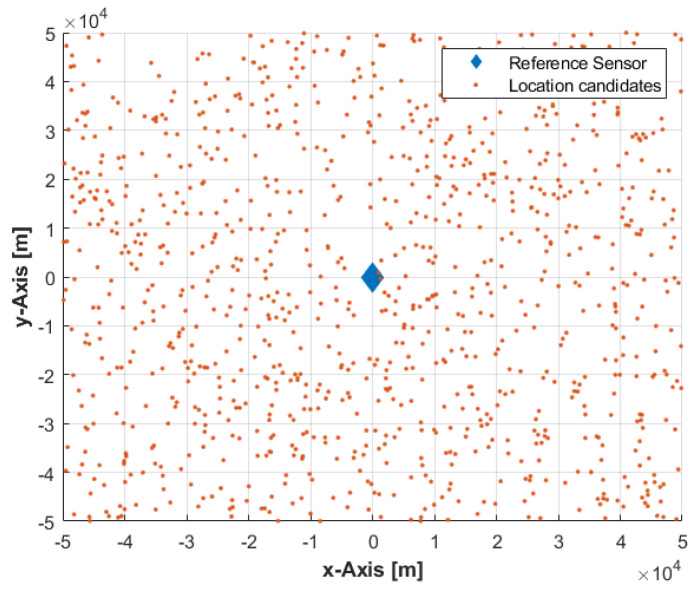
The set of uniformly distributed random emitter location candidates within the solution space corresponds to the reference sensor coverage.

The sample-based algorithm generates a set of p uniformly distributed random emitter location candidates, i.e., samples, over the area corresponding to the reference sensor coverage (see Figure 3) to approximate the solution space, S, in Equation (15) at any time instant according to:

(20) $$s_{j}\sim U\left(x_{1}-d,x_{1}+d\right)$$

where $x_{1}={\left[x_{1},y_{1}\right]}^{T}$ is the 2D location of the first sensor, i.e., reference sensor, and $d={\left[d_{x},d_{y}\right]}^{T}$ represents the coverage of the reference sensor in the *x* and *y* directions. In this work, it is assumed that $d_{x}=d_{y}=50\;\mathrm{km}$. Then, the sample-based algorithm continues to execute the procedures given in Equations (16)–(19), i.e., the only difference to the grid-based algorithm is the process of location candidate generation.

The results given by the proposed algorithms may be similar to the results one would expect from other prior art algorithms. However, the proposed algorithms have fewer requirements in terms of prior information about the distribution of measurement errors and the initial guess of emitter location. FDoA measurements of a radio frequency (RF) signal are assumed in the simulations. However, the developed algorithms are equally applicable to FDoA measurements of diverse signals.

Geographical information may also be included in the probability determination. For example, the probability would be equal to zero, i.e., impossible, for all emitter location candidates on the land mass if it is known that the emitter signal is sea-based. In certain circumstances, it would even be desired to adjust the grid resolution or the number of samples to allow faster or more accurate emitter location estimation. A coarse grid size or a low number of samples may be used for a rough emitter location estimation. Regions of higher probability may then be subdivided using a finer grid size, or a higher number of samples for a more accurate emitter location estimation. The operator, thus, can define the solution space and grid resolution or the number of samples according to the available resources and information (including own knowledge, experience, and judgment) to facilitate emitter location at the desired level of accuracy and speed of computation. The selected grid resolution or number of samples does not need to be much finer than the achievable accuracy as predicted by the CRLB. Trade-offs between computation time and memory storage size are commonly conducted during system definition and development phases.

Any PDF may be used as the measurement error model for a sensor pair. Gaussian measurement error models are assumed in this study, by way of example only, because they are the most common models adopted in practical applications, and to facilitate the CRLB benchmarking. Gaussian error models are developed by proper calibration of the sensor systems and, thus, are sufficient for geolocation. However, measurement error models need not be uniform for each sensor over the entire solution space. Therefore, the error model for a given sensor may be a set of different PDFs (or even bias values) applicable in different regions of the solution space or at different times/weather conditions.

## 4. Simulation Results

Computer simulations were conducted to corroborate the viability of the developed emitter location solutions and to gain insight into the working of the proposed algorithms. The selected simulation parameters are only notional examples for performance evaluation purposes and do not outline specific tasks or missions. However, the results may be useful for an operational context on use cases and requirements. Performance analysis relies on Monte Carlo simulations and on the CRLB, which is the most popular statistical performance bound by far. The FDoA measurements are converted into range rate difference measurements, which are generated according to Equation (8). The standard deviation of FDoA measurement noise or error is denoted $\sigma_{m}$.

Long-haul voice links can be found across ultra-high frequency (UHF) bands (300 MHz–1 GHz). Digital communications via datalink are spread across the low gigahertz spectrum, e.g., Link-16 occupies the L band (1–2 GHz). Accordingly, the emitter was assumed to be stationary, transmitting at a known fixed frequency of 1 GHz, and located on the surface of a flat area. Emitter location is accomplished using FDoA measurements from a single instant or single-time snapshot, i.e., the sensors can intercept the emitter frequency only once. Multiple-instant FDoA observations improve performance, since the sensors can collect more FDoA measurements over some time.

**Figure 4:**
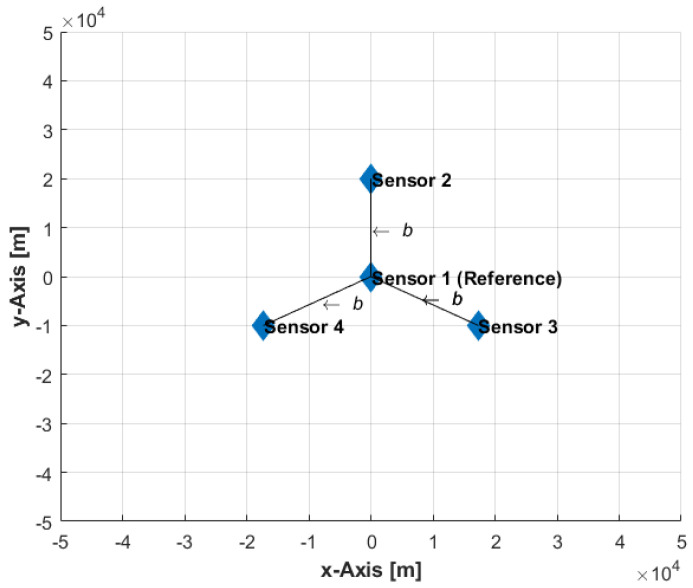
Formation of the sensors at the instant of measurements.

In the first set of simulations, 1000 independent stationary emitter locations have been generated by a uniform distribution within an area of 100 km×100 km at various measurement error conditions $\left(\sigma_{m}=1,\;5,\;\mathrm{and}\;10\;\mathrm{Hz}\right)$, sensor velocities (v=50, 100, and 150 m/s), and baselines (b=10, 15, and 20 km), where the baseline, b, is the equal spacing from reference sensor to the remaining sensors (see Figure 4). Four sensors on flying platforms (which could be manned or unmanned) collect single-time FDoA measurements from a radiating emitter. At the instant of measurements, the sensors are located at (0,0), (0,b), $\left(b\cos\left(-30^{{}^{\circ}}\right),b\sin\left(-30^{{}^{\circ}}\right)\right)$, and ($b\cos\left(210^{{}^{\circ}}\right),b\sin\left(210^{{}^{\circ}}\right)$), as illustrated in Figure 4, and all fly with a constant velocity of $v_{i}=\left(0,v\right)$. At each random emitter location, the grid-based and sample-based solution accuracies have been computed, with various grid resolutions (1000, 500, 200, and 100 m, which correspond to 10,201; 40,401; 251,001; and 1,002,001 location candidates, respectively) and a various number of samples (10^3^, 10^4^, 10^5^, and 10^6^), along with the corresponding CRLB. The performance is measured by averaging the results of the 1000 independent ensample runs and is given in terms of the circular error probable (CEP) computed from the MSE and CRLB. Thus, the set of these simulations averages the impact of the various sensors-emitter geometries on accuracy. However, the simulations reveal the general accuracy trends and impact on accuracy caused by varying the values of the investigated parameters.

**Figure 5:**
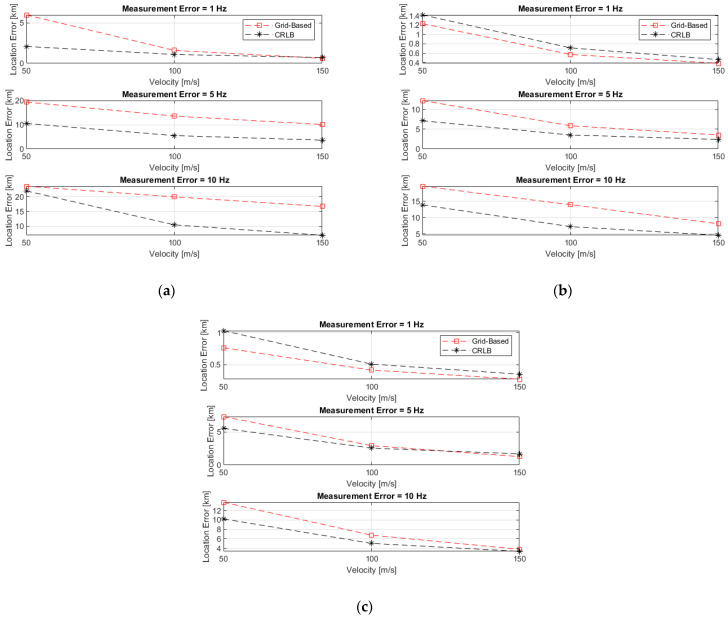
Grid-based solution accuracy with a grid resolution of 100 m and the corresponding CRLB, with various baselines of: (**a**) 10 km; (**b**) 15 km; (**c**) 20 km.

For brevity, Figure 5 depicts only the accuracy of the grid-based solution with a grid resolution of 100 m and the corresponding CRLB. Emitter location accuracy increases with higher velocities and longer baselines. The accuracy is sensitive to the level of measurement noise. Therefore, the performance worsens significantly at the higher measurement noise levels of 5 and 10 Hz. At more favorable conditions, i.e., a measurement noise level of 1 Hz and baselines of 15 and 20 km, the grid-based solution accuracy reaches beyond the less optimistic CRLB predictions because in such situations, the CRLB is non-strict due to the high nonlinearity of the problem.

Table 1 lists the accuracy of the grid-based and sample-based solutions considering a baseline of 20 km with various grid resolutions and a varied number of samples, respectively, at various sensor velocities and measurement noise levels, and the CRLB corresponding to each case. We can see that at less favorable conditions, i.e., measurement noise levels of 5 and 10 Hz and CRLB > 2 km, the performance of the grid-based solutions is generally similar because the achievable accuracy as predicted by the CRLB is much larger than the largest considered grid size of 1000 m. At more favorable conditions, i.e., CRLB < 2 km, the grid-based solutions remain generally similar and, in many situations, outperform the CRLB predictions because the CRLB is non-strict, as mentioned. The same observations apply to the sample-based solutions except with 10^3^ samples at the more favorable conditions. When the CRLB predicts an accuracy of less than 2 km, it would not be possible to use only 10^3^ samples to randomly generate an adequate number of samples close enough to the true emitter location to fully exploit the favorable situation. Results with baselines of 10 and 15 km showed similar trends and relative performances and, therefore, were omitted for brevity.

**Figure 6:**
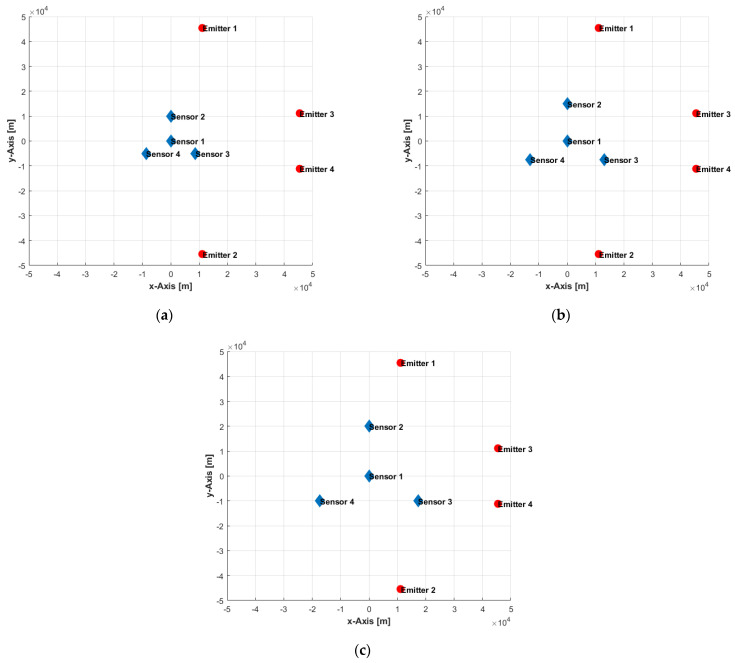
Sensors-emitters geometry with various baselines of: (**a**) 10 km; (**b**) 15 km; (**c**) 20 km.

In the second set of simulations, the sensors’ locations are kept unchanged, and four emitter locations are selected within the same area of 100 km×100 km to investigate the impact of sensors-emitter geometries on accuracy by various baselines (b=10, 15, and 20 km) (see Figure 6). Table 2 lists the achieved accuracy of the grid-based and sample-based solutions with a grid resolution of 100 m and 10^6^ samples, respectively, in addition to the corresponding CRLB, where the measurement noise $\sigma_{m}=1\;\mathrm{Hz}$ and all sensors fly with a constant velocity of $v_{i}=\left(0,150\right)\;m/s$. We can see that both solutions achieve similar accuracies and reach beyond the non-strict CRLB predictions. The only exception is at emitter location 4 with baseline b=20 km, where the performance degraded significantly, despite the optimistic prediction of the CRLB because the emitter, third sensor, and fourth sensor are quasi-collinear, as depicted in Figure 6c. Moreover, the CRLB computations depend on the Gaussian measurement errors assumption, whereas the high nonlinearity of the problem and the complex interdependences make the impact of measurement errors on the emitter location accuracy non-Gaussian and, thus, renders the CRLB predictions inaccurate. The results when applying various measurement error conditions, sensor velocities, grid resolutions, and a varied number of samples, as in the previous set of simulations, showed similar trends and, therefore, were omitted for brevity.

## 5. Conclusions

The radar uncertainty principle confirms that range and Doppler resolutions are inversely related. The evaluation of the CAF may be broad in the range or frequency direction, dependent on the type of received signal, making it difficult to accurately find the maximum (peak) of the cross-ambiguity surface corresponding to the TDoA or FDoA measurement. Thus, different signals will be more accurate in the TDoA or the FDoA measurements. Therefore, it is useful to investigate methods for emitter location using TDoA or FDoA measurements only.

This work developed algorithms and provided proof of concept for the emitter location problem using single-time FDoA measurements only, since it is less widely studied in the literature. Computer simulations were conducted to corroborate the viability of the developed algorithms/approach. The achieved accuracies are sufficient for early warning purposes, preparing defenses, and cueing more accurate location sensors by directing additional surveillance resources.

Since the FDoA-based emitter location problem is highly nonlinear, the solution is often ill-conditioned and sensitive to noise. Therefore, more investigations and tools are needed to better understand the complicated relationships between emitter-sensors geometries, velocities, and FDoA measurements.

The developed algorithms assumed a single emitter in isolation radiating a signal with a known constant carrier center frequency, perfect knowledge of the sensors’ locations and velocities, and uncorrelated measurement noise. Therefore, many extensions to more general problems can be applied to account for an unknown or erroneous carrier center frequency of the emitter’s signal, the presence of sensor location and velocity errors, correlated noise, moving emitter, multiple emitters, and the availability of successive-time FDoA measurements to develop tracking or target motion analysis (TMA) algorithms and to improve location accuracy.

## Figures and Tables

**Table 1:** Solution accuracies and corresponding Cramér–Rao lower bound (CRLB) with a baseline of 20 km.

		Grid-Based Accuracy (km)				Sample-Based Accuracy (km)				CRLB (km)
		Grid Resolution (m)				Number of Samples				
		1000	500	200	100	10^3^	10^4^	10^5^	10^6^	
σ_m_ = 1 Hz	v= 50 m/s	0.869	0.812	0.765	0.764	1.568	0.923	0.773	0.763	1.031
	v= 100 m/s	0.554	0.461	0.430	0.417	1.392	0.632	0.449	0.417	0.51
	v= 150 m/s	0.438	0.330	0.286	0.275	1.434	0.534	0.311	0.278	0.353
σ_m_ = 5 Hz	v= 50 m/s	7.279	7.384	7.354	7.296	7.049	7.226	7.431	7.369	5.52
	v= 100 m/s	2.836	2.897	2.914	2.924	3.320	2.810	2.872	2.923	2.55
	v= 150 m/s	1.356	1.301	1.277	1.275	1.953	1.374	1.294	1.281	1.687
σ_m_ = 10 Hz	v= 50 m/s	13.712	13.692	13.759	13.761	13.662	13.557	13.752	13.756	10.234
	v= 100 m/s	6.804	6.829	6.750	6.771	6.624	7.046	6.786	6.772	5.029
	v= 150 m/s	3.728	3.832	3.787	3.789	4.426	3.767	3.731	3.726	3.342

**Table 2:** Accuracy of grid-based (a grid resolution of 100 m) and sample-based (10^6^ samples) solutions vs. the CRLB at the selected four emitter locations with measurement noise of 1 Hz and sensor constant velocity of 150 m/s.

		*b* = 10 km	*b* = 15 km	*b* = 20 km
Location 1	Grid-Based Accuracy (km)	1.976	1.286	0.817
	Sample-Based Accuracy (km)	1.975	1.284	0.818
	CRLB (km)	2.612	1.671	1.088
Location 2	Grid-Based Accuracy (km)	1.712	0.659	0.339
	Sample-Based Accuracy (km)	1.709	0.663	0.341
	CRLB (km)	2.166	0.765	0.393
Location 3	Grid-Based Accuracy (km)	0.351	0.272	0.261
	Sample-Based Accuracy (km)	0.351	0.275	0.261
	CRLB (km)	0.474	0.355	0.305
Location 4	Grid-Based Accuracy (km)	0.329	0.259	13.213
	Sample-Based Accuracy (km)	0.329	0.259	13.281
	CRLB (km)	0.472	0.33	0.275

## Data Availability

Not applicable.

## Abbreviations

2D	Two-dimensional
3D	Three-dimensional
AoA	Angle of arrival
CAF	Cross-ambiguity function
CEP	Circular error probable
CRLB	Cramér–Rao lower bound
DD	Differential Doppler
DDR	Differential Doppler rate
DF	Direction finding
ES	Electronic support
EW	Electronic warfare
FDoA	Frequency difference of arrival
FIM	Fisher information matrix
MSE	Mean-square error
PDF	Probability density function
RF	Radio frequency
RMSE	Root-mean-square error
SNR	Signal-to-noise ratio
SWaP	Size, weight, and power
TDoA	Time difference of arrival
TMA	Target motion analysis
UAS	Unmanned aircraft system
UHF	Ultra-high frequency
VHF	Very-high frequency
